# Supplementary material for: Modeling glioblastoma heterogeneity as a dynamic network of cell states
Source: Mol Syst Biol. 2021 Sep 16;17(9):e10105. doi: 10.15252/msb.202010105 (PMC8444284; doi:10.15252/msb.202010105)
Supplement: Supplementary file 5 — Source Data for Figure 3 [file MSB-17-e10105-s001.zip › Figure3A_sourcedata/GSEA_3065/hallmarks_state4.GseaPreranked.1623416296068/HALLMARK_UV_RESPONSE_UP.html]

Details for gene set HALLMARK\_UV\_RESPONSE\_UP[GSEA]

|  || Dataset | state4 |
| Phenotype | NoPhenotypeAvailable |
| Upregulated in class | na\_neg |
| GeneSet | HALLMARK\_UV\_RESPONSE\_UP |
| Enrichment Score (ES) | -0.44611624 |
| Normalized Enrichment Score (NES) | -1.5407773 |
| Nominal p-value | 0.00698324 |
| FDR q-value | 0.043363374 |
| FWER p-Value | 0.403 |
Table: GSEA Results Summary

  

Fig 1: Enrichment plot: HALLMARK\_UV\_RESPONSE\_UP      
 Profile of the Running ES Score & Positions of GeneSet Members on the Rank Ordered List

  

| PROBE | GENE SYMBOL | GENE\_TITLE | RANK IN GENE LIST | RANK METRIC SCORE | RUNNING ES | CORE ENRICHMENT || 1 | ATP6V1F |  |  | 96 | 0.251 | 0.0144 | No |
| 2 | SELENOW |  |  | 122 | 0.237 | 0.0386 | No |
| 3 | CNP |  |  | 332 | 0.169 | 0.0249 | No |
| 4 | SQSTM1 |  |  | 390 | 0.155 | 0.0342 | No |
| 5 | JUNB |  |  | 490 | 0.139 | 0.0347 | No |
| 6 | BTG2 |  |  | 689 | 0.116 | 0.0164 | No |
| 7 | CDC34 |  |  | 745 | 0.109 | 0.0205 | No |
| 8 | FOS |  |  | 832 | 0.101 | 0.0186 | No |
| 9 | SLC6A8 |  |  | 926 | 0.093 | 0.0147 | No |
| 10 | ACAA1 |  |  | 986 | 0.089 | 0.0158 | No |
| 11 | CA2 |  |  | 1037 | 0.085 | 0.0178 | No |
| 12 | ATP6V1C1 |  |  | 1145 | 0.079 | 0.0100 | No |
| 13 | CDKN1C |  |  | 1328 | 0.070 | -0.0112 | No |
| 14 | E2F5 |  |  | 1405 | 0.066 | -0.0156 | No |
| 15 | YKT6 |  |  | 1491 | 0.062 | -0.0220 | No |
| 16 | DNAJB1 |  |  | 1527 | 0.061 | -0.0204 | No |
| 17 | LHX2 |  |  | 1534 | 0.061 | -0.0141 | No |
| 18 | PRKACA |  |  | 1769 | 0.053 | -0.0457 | No |
| 19 | GRINA |  |  | 1904 | 0.049 | -0.0615 | No |
| 20 | UROD |  |  | 1997 | 0.047 | -0.0707 | No |
| 21 | PDLIM3 |  |  | 2070 | 0.045 | -0.0770 | No |
| 22 | TST |  |  | 2118 | 0.043 | -0.0795 | No |
| 23 | MSX1 |  |  | 2143 | 0.043 | -0.0783 | No |
| 24 | CLCN2 |  |  | 2613 | 0.033 | -0.1502 | No |
| 25 | NPTXR |  |  | 2616 | 0.033 | -0.1467 | No |
| 26 | KLHDC3 |  |  | 2669 | 0.031 | -0.1514 | No |
| 27 | WIZ |  |  | 2676 | 0.031 | -0.1486 | No |
| 28 | CLTB |  |  | 2839 | 0.028 | -0.1715 | No |
| 29 | RHOB |  |  | 2911 | 0.026 | -0.1799 | No |
| 30 | MRPL23 |  |  | 2947 | 0.026 | -0.1825 | No |
| 31 | STK25 |  |  | 2956 | 0.026 | -0.1807 | No |
| 32 | FURIN |  |  | 3088 | 0.023 | -0.1991 | No |
| 33 | AP2S1 |  |  | 3110 | 0.023 | -0.1998 | No |
| 34 | AMD1 |  |  | 3213 | 0.021 | -0.2138 | No |
| 35 | IL6ST |  |  | 3218 | 0.021 | -0.2120 | No |
| 36 | PDAP1 |  |  | 3316 | 0.018 | -0.2256 | No |
| 37 | CDO1 |  |  | 3358 | 0.017 | -0.2302 | No |
| 38 | SLC25A4 |  |  | 3579 | 0.010 | -0.2647 | No |
| 39 | BID |  |  | 3898 | -0.003 | -0.3157 | No |
| 40 | NTRK3 |  |  | 3955 | -0.007 | -0.3239 | No |
| 41 | PPP1R2 |  |  | 4009 | -0.010 | -0.3313 | No |
| 42 | BTG1 |  |  | 4064 | -0.013 | -0.3384 | No |
| 43 | RAB27A |  |  | 4217 | -0.023 | -0.3603 | No |
| 44 | ATF3 |  |  | 4262 | -0.026 | -0.3643 | No |
| 45 | BMP2 |  |  | 4398 | -0.036 | -0.3819 | No |
| 46 | PPT1 |  |  | 4417 | -0.037 | -0.3804 | No |
| 47 | TFRC |  |  | 4532 | -0.047 | -0.3933 | No |
| 48 | CCND3 |  |  | 4578 | -0.050 | -0.3947 | No |
| 49 | DNAJA1 |  |  | 4635 | -0.054 | -0.3973 | No |
| 50 | TYRO3 |  |  | 4938 | -0.078 | -0.4368 | Yes |
| 51 | EIF5 |  |  | 4991 | -0.082 | -0.4355 | Yes |
| 52 | HSPA13 |  |  | 5007 | -0.083 | -0.4280 | Yes |
| 53 | CDK2 |  |  | 5015 | -0.084 | -0.4192 | Yes |
| 54 | CDC5L |  |  | 5049 | -0.087 | -0.4142 | Yes |
| 55 | CYB5B |  |  | 5098 | -0.092 | -0.4110 | Yes |
| 56 | PARP2 |  |  | 5119 | -0.093 | -0.4032 | Yes |
| 57 | POLR2H |  |  | 5127 | -0.094 | -0.3932 | Yes |
| 58 | PPAT |  |  | 5135 | -0.095 | -0.3830 | Yes |
| 59 | IRF1 |  |  | 5157 | -0.098 | -0.3748 | Yes |
| 60 | CCNE1 |  |  | 5218 | -0.103 | -0.3722 | Yes |
| 61 | EIF2S3 |  |  | 5304 | -0.110 | -0.3729 | Yes |
| 62 | PPIF |  |  | 5337 | -0.112 | -0.3647 | Yes |
| 63 | STIP1 |  |  | 5458 | -0.126 | -0.3691 | Yes |
| 64 | FKBP4 |  |  | 5480 | -0.129 | -0.3571 | Yes |
| 65 | POLE3 |  |  | 5481 | -0.129 | -0.3418 | Yes |
| 66 | RPN1 |  |  | 5678 | -0.156 | -0.3548 | Yes |
| 67 | CHRNA5 |  |  | 5708 | -0.161 | -0.3403 | Yes |
| 68 | HYAL2 |  |  | 5742 | -0.170 | -0.3254 | Yes |
| 69 | IGFBP2 |  |  | 5777 | -0.179 | -0.3096 | Yes |
| 70 | PSMC3 |  |  | 5831 | -0.190 | -0.2956 | Yes |
| 71 | TMBIM6 |  |  | 5855 | -0.196 | -0.2761 | Yes |
| 72 | BTG3 |  |  | 5895 | -0.206 | -0.2579 | Yes |
| 73 | HNRNPU |  |  | 6008 | -0.241 | -0.2474 | Yes |
| 74 | FEN1 |  |  | 6035 | -0.256 | -0.2211 | Yes |
| 75 | BSG |  |  | 6050 | -0.269 | -0.1913 | Yes |
| 76 | RFC4 |  |  | 6185 | -0.399 | -0.1655 | Yes |
| 77 | CTSV |  |  | 6204 | -0.456 | -0.1141 | Yes |
| 78 | CCK |  |  | 6228 | -0.515 | -0.0566 | Yes |
| 79 | GGH |  |  | 6232 | -0.522 | 0.0050 | Yes |
Table: GSEA details [plain text format]

  

Fig 2: HALLMARK\_UV\_RESPONSE\_UP: Random ES distribution      
 Gene set null distribution of ES for **HALLMARK\_UV\_RESPONSE\_UP**
